# How walkable are our cities for older adults? Mediation of comfort, convenience, and aesthetics

**DOI:** 10.12688/f1000research.161627.1

**Published:** 2025-02-27

**Authors:** Akshatha Rao, Rama Devi Nandineni

**Affiliations:** 1 Manipal School of Architecture & Planning, Manipal Academy of Higher Education, Karnataka, Manipal, 576104, India

**Keywords:** Aesthetics, Age-friendly urban policies, Built environment, Comfort, Community engagement programs, Government support for infrastructure policies, Older adults, Walkability

## Abstract

**Background:**

This study investigates the mediating role of comfort (CMT), convenience (CNV), and aesthetics (AST) in the relationship between the enablers of an age-friendly built environment for walkability (BEW) and outcomes such as increased physical activity level (IPL), increased socialization (ISL), and improved quality of life (IQL) in older adults. The research emphasizes the importance of creating age-friendly environments to support the well-being and quality of life of older adults, with implications for urban planners and policymakers to promote sustainable and inclusive design.

**Methods:**

The research follows a positivist paradigm using a quantitative approach and survey strategy with a cross-sectional design. A sample of 333 older adults was selected using the convenience sampling technique, and data were gathered through a self-administered questionnaire.

**Results:**

Findings from hypothesis testing indicate that, among the enablers, age-friendly urban design policies (AUP) are the most significant construct, positively impacting all three mediating variables. AST was found to have a significant positive effect on IPL and ISL, while CMT and CNV significantly influenced IPL and IQL, respectively.

**Conclusion:**

These results suggest implications for urban planners and policymakers to enhance the contribution of BEW toward IPL, ISL, and IQL for older adults. The research offers valuable insights for academics and practitioners, emphasizing the importance of sustainable design while ensuring inclusivity to promote the well-being and quality of life of older adults.

## Introduction

The study of the BEW has gained momentum globally in the context of promoting the health and wellbeing of older adults and meeting inclusivity demands. Urban areas that encourage walking are recognized as critical for IPL, ISL, and IQL for older adults. With the aging global population, urban planners and policymakers increasingly focus on creating BEW that supports independent and active lifestyles in older adults ( Bonaccorsi et al., 2020; Zhang & Kim, 2023). As the demand for AUP grows, the role of BEW is pivotal in offering older adults the required CMT, CNV, and AST through enjoyable spaces ideal for walkability ( Scharlach & Lehning, 2013). Thus, the need for a BEW design reflects an international agenda to increase the quality of life for aging populations in cities worldwide.

In India, the focus on BEW is gaining importance more than ever before, given the country’s rapidly growing elderly population and the challenges of urbanization. Inadequate pedestrian infrastructure, limited access to green spaces, and often unwalkable environments that can lead to social isolation and reduced physical activity have been identified as the major challenges to overcome in the context of older adults in India ( Che Had et al., 2023; Marston & Van Hoof, 2019). Indian urban centres require more adaptive strategies to cater to the mobility needs of older adults, promoting inclusive environments that address cultural and socioeconomic factors unique to this demographic. Research exploring BEW in India remains limited, highlighting an urgent need for studies that assess how BEW can be given face lift to support the walkability needs of Indian older adults ( Vikram et al., 2024; Guo et al., 2024).

Recent studies provide meaningful insights into the antecedents and outcomes of BEW in the context of the well-being of older adults. For example, studies indicate that CMT, CNV, and AST within BEW strongly influence physical activity and social interactions among seniors ( Askarizad et al., 2024; Ory et al., 2016). Furthermore, research by Abdelfattah and Nasreldin (2019) and Aghaabbasi et al. (2018) emphasizes the need for walkability audit tools and evaluation frameworks to assess and improve BEW effectively. Despite these advancements, there is a notable research gap in the literature focused on how the collective efforts of community engagement programs (CEPs), government policies on infrastructure (GPI), and AUP contribute to creating inclusive BEW for older adults in a developing country in general and India in particular.

Under the backdrop of these studies, the current research aims to address this gap by examining the significance of the mediating role of constructs—CMT, CNV, and AST—between GPI, CEP, and AUP as the enablers and IPL, ISL, and IQL as the outcomes to be achieved through the BEW. By identifying the constructs that significantly impact older adults’ quality of life through BEW, this research seeks to offer actionable insights for urban planners and policymakers, contributing to the creation of more inclusive and supportive environments for aging populations in India. Infrastructure policies dictate the design and maintenance of pathways, sidewalks, and pedestrian crossings, thus influencing the CNV with which individuals can navigate on foot ( Young et al., 2020). Policies promoting mixed-use development and higher density contribute to the creation of conveniently located amenities ( Cervero et al., 2003).

## Hypothetical model

Earlier studies have formed the basis for the development of the hypothetical model of this research. The following are the linkages between the study variables.

### Linkage of GPI with CMT, CNV, and AST

The interconnection between GPI and the CMT aspects of walkability is pivotal for fostering pedestrian-friendly BEW. Infrastructure policies have a profound impact on the physical layout and amenities that directly impact the CMT of individuals navigating on foot ( Fan & Cui, 2023). Well-designed and comfortable sidewalks, benches, and aesthetically pleasing landscaping contribute to the physical comfort of pedestrians and promote walkability ( Ali & Baper, 2023). Infrastructure policies, which dictate the design and maintenance of public spaces, sidewalks, and thoroughfares, have a profound influence on the AST of the BEW ( Mandeli, 2019). GPIs, which prioritize green spaces, street trees, and landscaping, not only contribute to the visual beauty of walkable areas but also provide shade and a sense of tranquility ( Elderbrock et al., 2020).

While these are the findings of earlier studies, there is no empirical proof to support these findings; hence, the following hypothesis is postulated.

H _1a_: There is a positive significant relationship between *government support for infrastructure* and *the comfort* aspects of walkability.

H _2a_: There is a positive significant relationship between *government support for infrastructure* and *convenience* aspects of walkability.

H _3a_: There is a positive significant relationship between *government support for infrastructure* and *aesthetic* aspects of walkability.

### Linkage of CEP with CMT, CNV, and AST

CEP serves as a conduit for understanding the diverse needs and preferences of residents, fostering a collaborative approach to enhancing the CMT of public spaces ( Bull et al., 2008). Through participatory planning processes and community-led initiatives, residents become active contributors to the design and activation of public spaces, ensuring that these areas align with their cultural, social, and recreational aspirations ( Vercher et al., 2021). CEPs serve as crucial platforms for fostering collaborative dialogue between community members and local authorities, enabling the identification of specific needs related to CNV in walkability ( Svara & Denhardt, 2010). Engagement programs often lead to the incorporation of culturally significant elements into the urban fabric, creating visually stimulating environments that celebrate diversity and enhance the overall aesthetic experience of walking ( Chan et al., 2012).

There are many such conceptualizations related to these two variables; however, the empirical proof is missing, and hence, the following hypothesis is postulated.

H _4a_: There is a positive significant relationship between *community engagement programs* and *comfort* aspects of walkability.

H _5a_: There is a positive significant relationship between *community engagement programs* and *convenience* aspects of walkability.

H _6a_: There is a positive significant relationship between *community engagement programs* and *aesthetic* aspects of walkability.

### Linkage of AUP with CMT, CNV, and AST

Comfortable walking experiences involve protection from harsh weather conditions, such as excessive sunlight or rain, and design elements such as trees, awnings, or covered walkways contribute to the overall comfort of pedestrians, allowing them to enjoy their walks in various weather conditions ( Rosenberg et al., 2013). Benches strategically placed along pathways not only provide physical comfort but also contribute to a sense of CMT, encouraging older adults to engage in outdoor activities confidently ( Finlay et al., 2015). AUP prioritizes the integration of green spaces, well-maintained landscapes, and public art, fostering an aesthetically pleasing environment that encourages individuals to engage in outdoor activities (Caronte-Veisz, 2022). The design of pathways, pedestrian zones, and street furniture is thoughtfully curated to blend functionality with beauty, creating visually attractive spaces that promote a sense of well-being ( King et al., 2020).

While these facts are established on the basis of observations through qualitative studies, there is no empirical proof for these findings; hence, the following hypothesis is postulated.

H _7a_: There is a positive significant relationship between *age-friendly urban design policies* and *comfort* aspects of walkability.

H _8a_: There is a positive significant relationship between *age-friendly urban design policies* and *convenience* aspects of walkability.

H _9a_: There is a positive significant relationship between *age-friendly urban design policies* and *aesthetic* aspects of walkability.

### Linkage of CMT with IPL, ISL and IQL

Physical comfort, derived from ergonomic spaces, cosy homes, and comfortable attire, directly influences an individual’s sense of relaxation and reduces physical stressors in daily life ( Heijs & Stringer, 1987). CMT extends to environmental aspects as well, encompassing surroundings that offer tranquility, access to nature, and amenities that enhance daily life ( Brochado & Pereira, 2017). Emotional CMT arising from positive social interactions and a welcoming atmosphere reduces feelings of isolation and anxiety and promotes a sense of security, creating an environment conducive to open communication and togetherness ( Khairy et al., 2023). Emotional CMT, derived from positive social interactions, supportive relationships, and a sense of togetherness, plays a crucial role in boosting morale, reducing feelings of isolation, and promoting emotional resilience ( Suaib, 2024).

While these facts are established on the basis of observations through qualitative studies, there is no empirical proof for these findings; hence, the following hypothesis is postulated.

H _10a_: There is a positive significant relationship between *comfort* and *increased physical activity levels* through walkability.

H _11a_: There is a positive significant relationship between *comfort* and *increased socialization* through walkability.

H _12a_: There is a positive significant relationship between *comfort* and *improved quality of life* through walkability.

### Linkage of CNV with IPL, ISL and IQL

Physical activity is often impeded by barriers such as accessibility challenges or inconvenience, particularly for the elderly ( Baert et al., 2011). The reciprocal relationship is evident, as increased CNV correlates with IPL, contributing to improved cardiovascular health, enhanced mobility, and overall well-being ( Chen et al., 2011). Age-friendly communities with conveniently located healthcare facilities, accessible public spaces, and easily navigable urban layouts contribute to reduced stress and IQL for older adults ( Marston & Van Hoof, 2019). Additionally, CNV transportation options, home modifications for accessibility, and user-friendly technologies further empower aged adults to engage in activities they value ( Dabelko-Schoeny et al., 2020).

While these facts are established on the basis of observations through qualitative studies, there is no empirical proof for these findings; hence, the following hypothesis is postulated.

H _13a_: There is a positive significant relationship between *convenience* and *increased physical activity levels* through walkability.

H _14a_: There is a positive significant relationship between *convenience* and *increased socialization* through walkability.

H _15a_: There is a positive significant relationship between *convenience* and *improved quality of life* through walkability.

### Linkage of the AST with the IPL, ISL and IQL

Aesthetically pleasing spaces, such as well-maintained parks, scenic walking paths, and thoughtfully landscaped areas, create an inviting atmosphere that encourages seniors to engage in outdoor activities ( Semenzato et al., 2011). Integrating art, greenery, and aesthetic elements into urban planning and recreational areas enhances overall ambiance, making physical activity more enticing for older adults ( Zhang & Kim, 2023). Aesthetically pleasing communal spaces, adorned with greenery, public art, and inviting architecture, create a welcoming atmosphere that can lead to ISL among older adults ( Cattell et al., 2008). AST, including the design of public spaces and community hubs, plays a crucial role in reducing feelings of isolation and loneliness, contributing to improved mental well-being for the elderly ( Leikas, 2020). The linkage between AST and an IQL through walkability for aged adults encapsulates the transformative impact of visually appealing and well-designed environments on their overall well-being ( Zhao & Chung, 2017). Aesthetically pleasing walkable spaces, characterized by charming landscapes, well-maintained pathways, and thoughtful urban design, not only motivate seniors to engage in regular physical activity but also contribute to increased enjoyment during their walks ( Khder et al., 2016).

While these facts are established on the basis of observations through qualitative studies, there is no empirical proof for these findings; hence, the following hypothesis is postulated.

H _16a_: There is a positive significant relationship between *aesthetics* and *increased physical activity levels* through walkability.

H _17a_: There is a positive significant relationship between *aesthetics* and *increased socialization* through walkability.

H _18a_: There is a positive significant relationship between *aesthetics* and *improved quality of life* through walkability.

**Figure 1.**
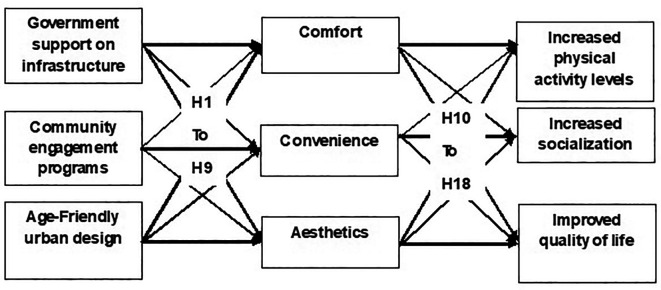
Hypothetical model.

The hypothetical model is shown in Figure 1.

## Methods

### Research Philosophy

This research is based on the positivist paradigm with a quantitative approach using a survey strategy. A self-administered questionnaire was used to collect quantitative data via both manual and electronic formats. The convenience sampling technique was used, as random selection through unique identifiers from a population size of 170,000 older adults from the sampling frame was not practicable, as demanded by probability sampling. Moreover, convenience sampling, particularly in the context of SEM analysis, is a widely accepted technique because it provides flexibility to maximize the sample through random selection from all seven taluks from the district (e.g., Mukuka, 2024). The analysis includes both descriptive statistics through a measurement model and inferential statistics through the structural model.

### The study area

The Udupi District in Karnataka, India, is the geographical location selected for this research, as it is a typical urbanizing study area in a rapidly developing country. It is also the most common type after the destinations of older adults because of its proximity to essential services, parks, shrines, recreational areas surrounded by nature with the Arabian Sea on one side, and the Western Ghats of India on the other. Moreover, Udupi is also a popular tourist and pilgrimage destination known for its temples, beaches, and historical monuments (Vikram, 2024).

### Research design

This research is a correlational study with a cross-sectional design and a quantitative approach. A self-administered questionnaire built on a 5-point Likert scale was used and analysed through SEM to determine the statistical significance of the relationships between the study variables. To ensure data reliability and validity in this type of research design, measures such as Cronbach’s alpha, composite reliability, discriminant validity, and Rho-A were employed. This methodological approach offers a robust research design for exploring how GPI, CEP, and AUP impact the mediation of CMT, CNV and AST issues related to walkability for older adults in the built environment.

### Sampling procedure

A sample of 333 older adults aged 60 years and above living in Udupi District was selected for this research via a convenience sampling technique. For the effect size (f ^2^) = 0.3 (medium effect) and alpha error probability (α) = 0.05, the G-Power (1 - β) was found to be 0.99, confirming the adequacy of the sample size. The sample was drawn from various BEW areas within Udupi districts, ensuring diverse representations of the age groups of older adults, genders, marital statuses, educational backgrounds, and socioeconomic statuses.

### Questionnaire design

The questionnaire design followed the standard procedure of selecting items from standard scales designed by earlier researchers. Items from the Urban Accessibility Index (UAI), Infrastructure Quality Index (IQI), and Sustainable Infrastructure Index (SII) ( Alderete. 2020; Benítez-Márquez et al., 2022; Lai & Cole, 2023) were chosen for GPI; items from the Community Engagement Scale (CES), Community Participation Scale (CPS), and Citizen Participation Assessment Tool (CPAT) ( Doolittle & Faul, 2013; Chipuer & Pretty. 1999) were chosen for CEP, and items from the WHO Age-Friendly Cities Questionnaire (WAFQ), Age-Friendly Communities Scale (AFCS), and Built Environment Assessment Tool (BEAT) ( Dikken et al., 2020; Glanz et al., 2016; Gibberd, 2019) were chosen for the AUG; the Pedestrian Comfort and Safety Assessment (PCSA) ( Asadi-Shekari et al., 2015) and Perceived Comfort Scale (PCS) ( Zegeer, 2006) were chosen for the CMT scale; the Pedestrian Convenience Sampling Assessment Tool (PCAT) ( Aghaabbasi et al., 2018) and Walkability Audit Tool (WAT) ( Soltani et al., 2018) were chosen for CNV; the Walkable Design Visual Quality Assessment (VQA) ( Abdelfattah & Nasreldin, 2019) was chosen for the AST scale; the International Physical Activity Questionnaire (IPAQ) ( Armstrong & Bull, 2006) was chosen for IPL; and the Neighborhood Social Cohesion Questionnaire (NSCQ) ( Agampodi et al., 2015) was chosen for ISL. The items in the questionnaire were modified slightly to suit the local context; hence, reliability and validity were reassessed through Cronbach’s alpha reliability measures.

The first section of the questionnaire was reserved for assimilating sociodemographic information, and the second section comprised 5-point Likert scale items to measure the perceptions of BEW by older adults of GPI, CEP, AUP, CMT, CNV, AST, IPL, ISL, and IQL.

### Data collection process

The data collection process was carried out between March and June 2024. The researcher personally collected hardcopies of the self-administered questionnaire filled out by participants in their homes, parks, and community centers across different locations in the Udupi district. The items in the questionnaire were briefly presented by the researcher to the participants, highlighting their importance during the personal model of data collection, and a brief explanation of the research was provided in the Google Form with the contact details of the researcher to revert back in case of any difficulty. Google forms were also distributed through contacts in community centres, old-age homes, health care centres, and recreation clubs. While 186 hard copies were collected through hardcopies of the completed questionnaire, the remaining 147 were obtained through Google Forms.

### Ethical considerations

The study was conducted in accordance with ethical guidelines, with the rights and well-being of the participants being protected. Informed consent was obtained from all participants before the commencement of data collection. Anonymity and the confidentiality of the responses were declared in the questionnaire, which communicated well to all the respondents. They were informed that their participation was voluntary and that they could withdraw from the study at any point without any consequences. The Institutional Ethics Committee (IEC) had formally given ethical clearance for the conduct of this research, having ratified the entire research procedure.

## Results and analysis

### Sociodemographic characteristics of the samples

Table 1 shows a nearly equal gender distribution, with 52.9% male and 47.1% female respondents observed in the sample selected for this research. The age group of 60–65 years (45.0%) was the majority, followed by the age group of 65–70 years (40.9%). Among the respondents, the majority were married (41.5%), the next in the line widowed (26.1%), followed by unmarried (16.8%), with representations from other categories such as divorced (6.9%), separated (6.6%), and in the ‘other’ category (2.1%), which includes live-in relationships. Furthermore, slightly more than half of the respondents (51.3%) had a bachelor’s degree, followed by a master’s degree (26.4%), and a smaller percentage had diplomas (14.5%), while a small percentage had certificates (3.6%) or doctor of philosophy degrees (3.6%). Finally, the majority of participants (47.8%) belonged to middle-income group-1 (7,150–14,300 USD per annum), followed by middle-income group-2 (34.2%) (14,300–21,450 USD per annum), low-income group (11.7%) (3,565–7,150 USD per annum), and high-income group (6.3%) (greater than 21,450 USD per annum), with a small number (4.2%) below the poverty line (less than 3,565 USD per annum).

**Table 1.** Sociodemographic characteristics of the sample (N=333).

Characteristics	Number	Percentage
**Gender**		
Male	176.0	52.9
Female	157.0	47.1
**Age**		
Above 60 up to 65 years	150	45.0
Above 65 up to 70 years	136	40.9
Above 70 up to 75 years	24	7.2
Above 75 up to 80 years	20	6.0
Above 85 years	3	0.9
**Marital Status**		
Unmarried	56	16.8
Married	138	41.5
Divorced	23	6.9
Widowed	87	26.1
Separated	22	6.6
Other	7	2.1
**Educational qualification**		
Certificate	12	3.6
Diploma	48	14.5
Bachelor’s degree	172	51.3
Master’s degree	87	26.4
Ph.D. degree	12	3.6
Other	2	0.6
**Socioeconomic status**		
Below Poverty Line	14	4.2
Lower Income Group	25	7.5
Middle Income Group-1	159	47.8
Middle Income Group-2	114	34.2
High Income Group	21	6.3

### Measurement model

**Figure 2.**
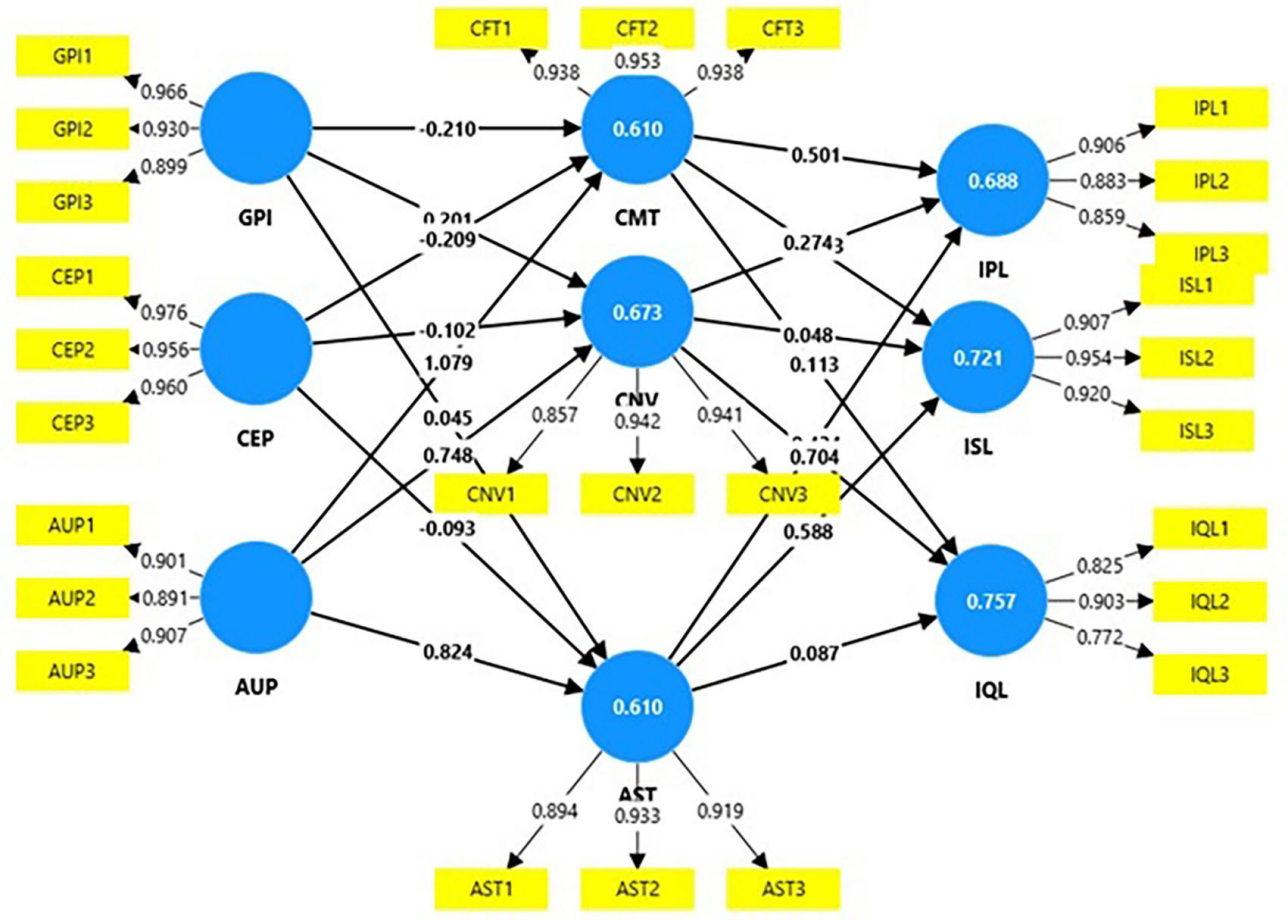
Measurement model.

The reliability and validity measures in Table 2 demonstrate a high level of internal consistency and construct validity for all the variables, with Cronbach’s alpha values for all the constructs above 0.70, with most values exceeding 0.90, indicating high internal reliability, and the composite reliability scores for all the constructs also surpass 0.90, confirming the reliability of the construct (cut-off of 0.7 for both; McNeish, 2018). Internal consistency is also measured through Rho_A values, which in this research range from 0.79--0.96, demonstrating a high to very high level of acceptability (cut-off=0.7; Trejo et al., 2024). The factor loadings were well above 0.70 ( Table 2 and Figure 2), suggesting that each item strongly represents its respective construct (cut-off=0.7; Kamranfar et al., 2023). The average variance extracted (AVE) values were significantly greater, indicating that the variance captured by the construct’s items was sufficiently high, with minimal error (cut-off=0.5; Cheung et al., 2024).

**Table 2.** Reliability and validity measures.

Construct	Items	Factor loadings	Cronbach's alpha	Rho_a	Composite reliability	Average variance extracted (AVE)
AST	AST1	0.89	0.90	0.91	0.94	0.84
	AST2	0.93				
	AST3	0.92				
AUP	AUP1	0.90	0.88	0.88	0.93	0.81
	AUP2	0.89				
	AUP3	0.91				
CEP	CEP1	0.98	0.96	0.96	0.98	0.93
	CEP2	0.96				
	CEP3	0.96				
CMT	CMT1	0.94	0.94	0.94	0.96	0.89
	CMT2	0.95				
	CMT3	0.94				
CNV	CNV1	0.86	0.90	0.91	0.94	0.84
	CNV2	0.94				
	CNV3	0.94				
GPI	GPI1	0.97	0.92	0.92	0.95	0.87
	GPI2	0.93				
	GPI3	0.90				
IPL	IPL1	0.91	0.86	0.86	0.91	0.78
	IPL2	0.88				
	IPL3	0.86				
IQL	IQL1	0.83	0.78	0.79	0.87	0.70
	IQL2	0.90				
	IQL3	0.77				
ISL	ISL1	0.91	0.92	0.92	0.95	0.86
	ISL2	0.95				
	ISL3	0.92				

AST: Aesthetics; AUP: Age friendly urban design policies; BEW: Built environment for walkability; CEP: Community engagement program; CMT: Comfort; CNV: Convenience; GPI: Government support for infrastructure policies; IPL: Increased physical activity; IQL: Improved quality of life; ISL: Increased socialization.

Table 3 demonstrates discriminant validity, which confirms that each construct is distinct from the rest of the constructs. The square roots of the AVE values (shown on the diagonal in bold) for each construct are all greater than the correlations with other constructs, indicating adequate discriminant validity. This pattern across all the constructs confirms that each construct in the model uniquely measures its intended concept, supporting the robustness of the measurement model. Thus, the indices in the measurement model confirm that the reliability and validity of the data and the questionnaire, respectively, are acceptable and that the data may be subjected to structural model analysis for hypothesis testing.

**Table 3.** Discriminant validity.

	AST	AUP	CEP	CMT	CNV	GPI	IPL	IQL	ISL
**AST**	**0.92**								
**AUP**	0.87	**0.91**							
**CEP**	0.67	0.91	**0.96**						
**CMT**	0.78	0.83	0.57	**0.94**					
**CNV**	0.89	0.91	0.72	0.87	**0.91**				
**GPI**	0.63	0.81	0.76	0.46	0.74	**0.93**			
**IPL**	0.85	0.75	0.55	0.86	0.80	0.42	**0.88**		
**IQL**	0.86	0.87	0.76	0.86	0.90	0.73	0.87	**0.83**	
**ISL**	0.90	0.80	0.59	0.79	0.82	0.55	0.84	0.82	**0.93**

AST: Aesthetics; AUP: Age friendly urban design policies; BEW: Built environment for walkability; CEP: Community engagement program; CMT: Comfort; CNV: Convenience; GPI: Government support for infrastructure policies; IPL: Increased physical activity; IQL: Improved quality of life; ISL: Increased socialization.

Figure 2 depicts path coefficients. This is the strength and direction of the relationships between the constructs in the model. AUP has a strong positive influence on AST, with a path coefficient of 0.82, indicating that improvements in AUP likely enhance perceptions of AST. On the other hand, CEP shows weak and negative associations with AST (-0.09), CMT (-0.21), and CNV (-0.10), indicating that these programs may not effectively support perceptions of aesthetics, comfort, or convenience. AST positively impacts ISL, with a coefficient of 0.59, and IPL, with a coefficient of 0.43, indicating that safety perceptions are influential in promoting socialization and physical activity. Finally, CNV has a notably strong positive relationship with IQL (0.70), suggesting that convenience is critical in enhancing the quality of life for older adults in this model.

### Structural model

**Figure 3.**
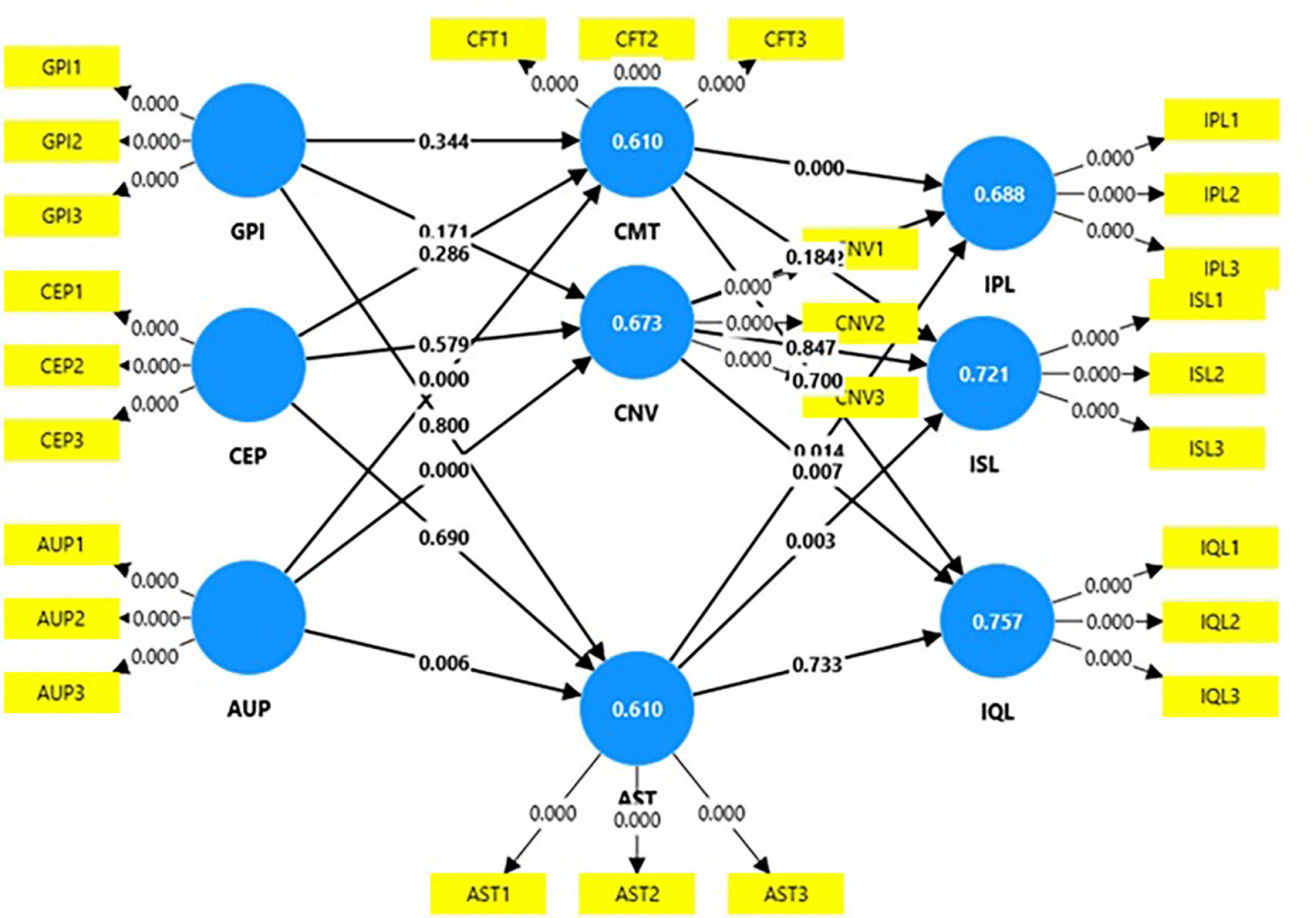
Structural model.

Table 4 shows that AUP has a positive significant relationship with AST (t=2.72; p=0.01), CMT (t=5.87; p=0.00), and CNV (t=4.62, p=0.00); AST has a positive significant relationship with IPL (t=2.45; p=0.01) and ISL (p=2.93; t=0.00); CMT has a positive significant relationship with IPL (t=3.56; t=0.00); and CNV has a positive significant relationship with IQL (t=2.70; p=0.01). Thus, the following hypotheses are supported ( Table 4, Figure 3):

**Table 4.** Hypothesis testing.

	Original sample (O)	Sample mean (M)	Standard deviation (STDEV)	T statistics (|O/STDEV|)	P values	Hypothesis
** AST -> IPL**	0.43	0.42	0.18	2.45	** 0.01**	** Supported**
AST -> IQL	0.09	0.04	0.25	0.34	0.73	Unsupported
**AST -> ISL**	0.59	0.58	0.20	2.93	**0.00**	**Supported**
**AUP -> AST**	0.82	0.73	0.30	2.72	**0.01**	**Supported**
**AUP -> CMT**	1.08	1.04	0.18	5.87	**0.00**	**Supported**
**AUP -> CNV**	0.75	0.79	0.16	4.62	**0.00**	**Supported**
CEP -> AST	-0.09	-0.03	0.23	0.40	0.69	Unsupported
CEP -> CMT	-0.21	-0.19	0.20	1.07	0.29	Unsupported
CEP -> CNV	-0.10	-0.12	0.18	0.56	0.58	Unsupported
**CMT -> IPL**	0.50	0.53	0.14	3.56	**0.00**	**Supported**
CMT -> IQL	0.11	0.14	0.29	0.39	0.70	Unsupported
CMT -> ISL	0.27	0.31	0.21	1.33	0.18	Unsupported
CNV -> IPL	-0.04	-0.06	0.18	0.24	0.81	Unsupported
**CNV -> IQL**	0.70	0.73	0.26	2.70	**0.01**	**Supported**
CNV -> ISL	0.05	0.01	0.25	0.19	0.85	Unsupported
GPI -> AST	0.05	0.09	0.18	0.25	0.80	Unsupported
GPI -> CMT	-0.21	-0.18	0.22	0.95	0.34	Unsupported
GPI -> CNV	0.20	0.17	0.15	1.37	0.17	Unsupported

AST: Aesthetics; AUP: Age friendly urban design policies; BEW: Built environment for walkability; CEP: Community engagement program; CMT: Comfort; CNV: Convenience; GPI: Government support for infrastructure policies; IPL: Increased physical activity; IQL: Improved quality of life; ISL: Increased socialization.

H _7a_: There is a positive significant relationship between *age-friendly urban design policies* and *comfort* aspects of walkability.

H _8a_: There is a positive significant relationship between *age-friendly urban design policies* and *convenience* aspects of walkability.

H _9a_: There is a positive significant relationship between *age-friendly urban design policies* and *aesthetic* aspects of walkability.

H _10a_: There is a positive significant relationship between *comfort* and *increased physical activity levels* through walkability.

H _15a_: There is a positive significant relationship between *convenience* and *improved quality of life* through walkability.

H _16a_: There is a positive significant relationship between *aesthetics* and *increased physical activity levels* through walkability.

H _17a_: There is a positive significant relationship between *aesthetics* and *increased socialization* through walkability.

The rest of the hypotheses are unsupported.

## Discussions

The findings of this research support the hypotheses that AUP has a positive and significant relationship with CMT, CNV, and AST in walkability. Scott (2021) emphasized that age-friendly urban policies, such as wider pathways, frequent resting areas, and adequate seating, significantly contribute to the comfort of older adults in public spaces. For older adults to engage more actively, CMT is essential in fostering a walkable environment that encourages them with their surroundings, enhancing their mobility and quality of life. Rui and Othengrafen (2023) reported that policies aimed at improving accessibility features such as pedestrian crossings, clear signage, and ramp installations make urban environments more navigable, accessible and CNV for older adults. These convenience features, through age-friendly policies, reduce mobility barriers, enabling older adults to move about with ease, which enhances their independence and overall well-being. The positive relationship between AUP and AST in walkability is also consistent with previous research, such as Senetra et al. (2024), who reported that well-designed, aesthetically pleasing urban environments are associated with higher levels of satisfaction among older adults. Green spaces, public art, and aesthetically maintained walkways enhance the visual appeal of neighborhoods and contribute to the psychological well-being of older adults by making public spaces more inviting and enjoyable for leisurely walks and social interactions. Collectively, these findings underscore the importance of implementing AUP that prioritizes CMT, CNV, and AST in urban design to foster walkable environments conducive to active aging.

The finding that CMT in urban design positively affects IPL among older adults corroborates well with some previous studies highlighting the importance of comfortable environments in promoting active lifestyles in older adults. For example, Guo et al. (2024) surmised that comfortable, well-maintained walking paths with adequate resting areas encouraged older adults to engage more frequently in outdoor activities and helped them compensate for the sedentary lifestyle. Salvo et al. (2018) reported that BEW with resting options, even surfaces, and shade provide a CMT, making walkability more appealing and accessible for older adults. However, in contrast, some studies have shown that CMT alone may not fully address the walkability needs of older adults. For example, Sugiyama et al. (2023) argued that while CMT plays a role, it must be supported by other elements, such as proximity to amenities and community engagement opportunities, to sustain higher activity levels among this population. The current findings reinforce that CMT is a foundational requirement, as it removes physical and psychological barriers to activity, even if it may not be the only motivating factor. Furthermore, this study extends the research by showing that CMT not only increases activity levels but also sustains them by fostering a supportive environment for repeated use.

Hypothesis testing also revealed a significant positive relationship between CNV and IQL through walkability. This finding aligns well with earlier research that underscores CNV as a crucial factor for enhancing older adults’ well-being. For example, studies by Ory et al. (2016) indicate that accessible, convenient pathways encourage more frequent walking, which contributes to better physical and mental health outcomes. Similarly, Che Had et al. (2023) reported that well-planned, convenient urban infrastructure positively influences the mobility and autonomy of older adults, further supporting the link to an improved quality of life.

In contrast, however, research by Du et al. (2022) suggests that convenience alone may not always lead to quality-of-life improvements if other factors, such as safety or social support, are lacking. Nevertheless, many other studies, including those by Khamaj & Ali (2024), surmise that features such as nearby amenities, easy-to-navigate routes, proper signage, and accessible facilities collectively enhance the living experience of older adults by making essential services more reachable. Moreover, convenient environments reduce physical and psychological barriers to outdoor activity ( Guo et al., 2024). Thus, CNV in walkable urban design directly correlates with increased life satisfaction and IQL among older adults.

The findings from the hypothesis testing confirm that AST plays a significant role in both IPL and ISL through BEW, aligning with previous research emphasizing the impact of visually pleasing surroundings. Finlay et al. (2015) demonstrated that aesthetically pleasing landscapes, such as greenery, flower beds, and neat-looking pathways, encourage individuals to spend more time walking, as they find the environment more inviting and enjoyable for physical activities. Additionally, contrasting research by Sundling and Jakobsson (2023) suggests that while AST enhances walkability, it may sometimes have a lesser influence than other factors, such as proximity to amenities or safety, highlighting that the effect of aesthetics can vary by context. However, appealing design elements have the potential to create a sense of relaxation and motivation for activity ( Sugiyama et al., 2023), a finding reflected in the current results.

The relationship between AST and ISL is also corroborated by studies from Sugiyama et al. (2023), who reported that visually attractive environments attract more walkers and provide spaces where people are more inclined to interact with others. Bonaccorsi et al. (2020) suggested that greenery and visually appealing environments can have potential indirect effects on socialization, as attractive public spaces may encourage older adults to spend more time outdoors. Lak et al. (2020) revealed that the urban landscape and environmental cleanness, which are aspects of AST, contribute to creating environments that older adults find appealing and conducive to their socialization.

In contrast, research by Askarizad et al. (2024) suggests that while ASTs support socialization, other aspects, such as the availability of benches or seating areas, could be equally crucial, emphasizing that aesthetic appeal alone may not fully drive social interactions. However, the findings of the current research underscore that well-designed, attractive spaces foster a sense of community and sociability, as people tend to linger longer and connect with others when their surroundings are pleasant, as found by other researchers (e.g., Chang et al., 2024; Yang et al., 2024).

### Practical implications

The practical implications of this research are for policy makers and urban designers in the form of suggestions to encourage the walkability of older adults. The specific implications are as follows.

1. First, AUP had a significant positive association with CMT, CNV, and AST. Policymakers may prioritize age-friendly elements in urban design by incorporating CMT-enhancing urban spaces that include resting spots, benches with backrests and armrests, shaded areas, and well-maintained paths. These features make walking pathways more comfortable for older adults, encouraging their regular use. Urban designers may consider integrating frequent resting points along popular walkways and ensuring even surfaces to minimize tripping hazards, aligning with older adults’ mobility needs.
  To increase convenience in walkable environments, urban planners should ensure that pedestrian pathways are accessible, safe, and easily interconnected. This can be facilitated through clear signage, ramps, accessible pedestrian crossings, and wide sidewalks to support easy navigation for older adults. Policies should focus on the provision of public restrooms and hydration stations at regular intervals, making outdoor activities more feasible and worthwhile for older individuals.
  AUPs may focus on creating visually appealing and well-landscaped areas that include parks, lakes, and public art installations. Urban designers may focus on landscaping with native plants, clean walkways, water features, and colorful, well-maintained public spaces to improve the AST of the BEW. Such enhancements cause older adults to spend more time outdoors, promoting both mental and physical well-being.
  Policymakers should adopt frameworks that promote inclusive and age-friendly urban standards in urban development projects. Clear guidelines to promote CMPt, CNV, and AST specifically tailored to the needs of older adults must be developed. Regular feedback from CEP can further refine these frameworks, ensuring that urban spaces evolve in line with the preferences of the aging population.
2. On the basis of the finding that CMT has a significant positive relationship with IPL through walkability, policymakers and urban designers should consider several practical steps to further increase walkability for older adults.
  By creating shaded walkways and adding weather shelters, such as covered benches, urban spaces become more comfortable for older adults and can increase outdoor comfort for older adults, encouraging them to stay active even in diverse weather conditions.
  Using slip-resistant, cushioned materials for pathways and sidewalks to minimize fall risk has been used in several countries to enhance CMT successfully, and it has improved walking comfort, particularly for older adults walking with mobility aids, which may be attempted by urban designers. Ensuring evenness of the surfaces also provides stability of the BEW and enhances confidence among older adults for increased physical activity.
  Integrating restorative natural elements such as adding small garden patches, fountains, and greenery along walking routes can offer comfortable environments that encourage longer and more frequent walks of older adults.
  The installation of low-impact fitness equipment designed for older adults along walkable routes to offer convenient spots for light exercise may be considered by planners. This can further encourage physical activity and increase confidence in safe, age-appropriate exercises.
  The development of multiuse paths with safe buffer zones with designated lanes for pedestrians, cyclists, and mobility aids is also a worthwhile addition to BEW to promote CMT and encourage the physical activity levels of older adults. This approach has advantages such as minimizing congestion and making the BEW more accessible for older adults to stay active without fear of collisions.
3. As hypothesis testing revealed a significant positive relationship between CNV and IQL through walkability, there is a need to design urban BEW with essential amenities, such as grocery stores, medical facilities, and community centers, within walking distance. This would allow older adults to access vital services without extensive travel, enhancing both their independence and their daily life satisfaction.
  Urban designers may consider the installation of clear, age-friendly signage along pedestrian routes to help older adults navigate easily. Strategic placement of the signage and making it legible will also be important. It is necessary to include visual cues to reduce navigational stress and promote greater confidence in older adults in outdoor spaces.
  The construction of pedestrian pathways that are free of obstructions with even surfaces to accommodate all levels of mobility may also be prioritized. Accessible, well-maintained pathways can encourage older adults to walk more often, promoting physical health and autonomy, as revealed through hypothesis testing; hence, this becomes important.
  Placing seating arrangements and provisions for buying essentials at regular intervals along walkable routes to provide convenient rest stops is also worth considering. This design allows older adults to walk comfortably at their own pace, encouraging longer, more frequent walks with adequate resting in between.
  Finally, designing walkable urban hubs where older adults can socialize and participate in community activities may also be considered more seriously. Placing multiple services and recreational facilities within a central, accessible area enhances CNV and fosters social engagement, both of which contribute to improved quality of life.
4. AST plays a positive significant role in both IPL and ISL through BEW; hence, the following implications may be considered by policy makers and urban designers.
  AST can be improved by the presence of trees, plants, and landscaped areas along pedestrian routes with natural aesthetics, such as greenery and flower beds. All these factors can make BEW more visually appealing and encourage older adults to spend more time outdoors, supporting both IPL and ISL.
  The integration of art installations, murals, and sculptures along walkable paths and in public spaces may be considered by urban designers. Art in the urban landscape not only beautifies the environment but also provides conversation starters and gathering points, fostering social interaction among older adults.
  Placing fountains or small water features in parks and along walkable routes improved the AST. The calming effect of water elements can enhance the appeal of outdoor spaces, encouraging older adults to walk more and socialize in these pleasant surroundings.
  The design of sensory gardens with aromatic plants, varied textures, and visually stimulating colors may be considered by urban designers. These gardens encourage both physical activity and relaxation while encouraging social gatherings and interactions within a comfortable, aesthetically pleasing environment.
  The planning of landscaping that incorporates seasonal plants and decorations to maintain an attractive, ever-changing environment year-round has been attempted very successfully in several countries and may be considered by urban designers. Seasonal aesthetics give older adults a reason to return frequently, supporting continuous physical activity, and this will also improve their socialization.
  Placing aesthetically pleasing benches and seating areas that are visually integrated with the surroundings may also be considered by urban designers. Attractive and ergonomically designed seating areas can attract older adults to take a break during walks, rest, and engage in conversations, promoting socialization within a visually welcoming environment.

## Conclusion

This research is about BEW with an orientation towards tailoring it to the specific needs of older adults, an increasingly essential goal as urban areas globally, particularly in developing countries, face rapid urbanization. The study emphasized epistemological aspects of the BEW, such as CMT, CNV, and AST, impacting IPL, ISL, and IQL for older adults. Given the growing aged population in India, building a congenial environment that supports mobility, health, and social inclusion is of paramount importance for achieving sustainable urban living.

The research with a quantitative approach, grounded in a positivist paradigm, made use of a cross-sectional design with a survey strategy. Data were collected from a sample of 333 older adults through a self-administered questionnaire assessing variables related to urban design and walkability. The data were analysed via SEM to identify and quantify the significance of the relationships between key urban design features and the outcomes of interest in this research.

Hypothesis testing revealed that among the three enablers of this research, AUP was the only construct that had a positive significant effect on all three mediators, namely, CMT, CNV, and AST. AST was positively and significantly related to IPL and ISL, whereas CMT was positively and significantly related to IPL, and CNV was positively and significantly related to IQL. This emphasized the need for age-friendly urban policy design interventions that prioritize visually appealing, accessible, and comfortable environments. For policymakers and urban designers, these results highlight the value of integrating shaded walkways, seating areas, clear signage, and aesthetically pleasing landscapes to encourage outdoor activity and social interaction among older adults. The study suggests that targeted urban design improvements can effectively promote active, healthy aging by making neighborhoods more walkable and socially engaging, ultimately enhancing older adults’ well-being and autonomy.

The cross-sectional design is a major limitation of this research in terms of its ability to assess causal relationships, and the use of convenience sampling may restrict the generalizability of the findings to broader populations. Thus, future studies may consider longitudinal designs to track changes over time and assess the sustained impact of urban design improvements on older adults’ well-being through BEW. Expanding the geographic scope beyond a single district could also offer deeper insights.

In conclusion, this research is timely, especially for developing countries such as India, which has a rapidly aging population. As these nations face the dual challenge of urbanization and population aging, adopting AUP becomes crucial for fostering inclusive, sustainable cities that support the health, independence, and quality of life of their older adults.

## Statements and declaration

Not applicable.

## Ethical considerations

The study has been conducted in accordance with ethical guidelines with rights and well-being of the participants being protected. Kasturba Medical College institutional and Kasturba Hospital (constituent unit of Manipal Academy of Higher Education, Manipal-576104), Ethics Committee (Registration No. ECR/146/Inst/KA/2013/RR-19) (DHR Registration No. EC/NEW/INST/2019/374), formally gave ethical clearance for the conduct of this research having ratified the entire procedure of research with approval number -IEC1: 387/2022. Approval is valid from 5 ^th^ July 2023 to 31 ^st^ May 2027.

## Consent to participate

Written informed consent was obtained from all participants. Physical written consent was collected on paper to ensure that all participants fully understood the purpose of the study, procedures and any potential risks involved. Further, anonymity and the confidentiality of the responses was declared in the questionnaire and well communicated to all the respondents. They were informed that their participation was voluntary and that they could withdraw from the study at any point without any consequences.

## Data Availability

**The project contains the following underlying data:** The project contains following data Data Set- Questionnaire Survey Dataset Answers to questionnaire- Anonymised answers to the questionnaire: Figshare.com: HOW WALKABLE ARE OUR CITIES FOR OLDER ADULTS? MEDIATION OF COMFORT, CONVENIENCE, AND AESTHETICS, 10.6084/m9.figshare.28350671 Rao, Akshatha; Nandineni, Rama Devi (2025). The project contains following extended data: Questionnaire used for the survey: Figshare.com: HOW WALKABLE ARE OUR CITIES FOR OLDER ADULTS? MEDIATION OF COMFORT, CONVENIENCE, AND AESTHETICS, 10.6084/m9.figshare.28350671 Rao, Akshatha; Nandineni, Rama Devi (2025). Data are available under the terms of the Creative Commons Attribution 4.0 International license (CC-BY 4.0).
