# Supplementary material for: How walkable are our cities for older adults? Mediation of comfort, convenience, and aesthetics
Source: F1000Res. 2026 Jan 16;14:242. Originally published 2025 Feb 27. [Version 3] doi: 10.12688/f1000research.161627.3 (PMC12784048; doi:10.12688/f1000research.161627.3)
Supplement: Peer review history

# Reviewer report: Reviewer response for version 3

**DOI:** 10.5256/f1000research.195070.r450487

**Published:** 2026-01-29

**Authors:** Maria Teresa Baquero Larriva

**Affiliations:** 1 Universidad Politécnica de Madrid, Madrid, Spain

The authors have responded to my suggestions, and I believe that the article presents interesting data on a relevant topic that is worth disseminating. Although it has some limitations, which have been mentioned, I consider it indexed in its current state.

Is the work clearly and accurately presented and does it cite the current literature?

Yes

If applicable, is the statistical analysis and its interpretation appropriate?

Partly

Are all the source data underlying the results available to ensure full reproducibility?

Partly

Is the study design appropriate and is the work technically sound?

Yes

Are the conclusions drawn adequately supported by the results?

Partly

Are sufficient details of methods and analysis provided to allow replication by others?

Yes

Reviewer Expertise:

Urban planning, sustainability, age-friendly cities, urban health, accessibility, thermal comfort, environmental quality.

I confirm that I have read this submission and believe that I have an appropriate level of expertise to confirm that it is of an acceptable scientific standard.

# Reviewer report: Reviewer response for version 2

**DOI:** 10.5256/f1000research.185485.r441034

**Published:** 2026-01-08

**Authors:** Maria Teresa Baquero Larriva

**Affiliations:** 1 Universidad Politécnica de Madrid, Madrid, Spain

This study focuses on the built environment for walkability, with an emphasis on customizing it to the unique requirements of senior citizens, a goal that is becoming increasingly important as urban areas around the world, especially in developing nations, experience rapid urbanization.

 The study examines the epistemic features of the built environment, including walkability, which encompasses comfort, convenience, and aesthetics, and how these factors influence higher levels of physical activity, socialization, and quality of life among older adults. Building a welcoming environment that promotes mobility, health, and social inclusion is crucial for achieving sustainable urban living, particularly in light of India’s aging population.

 I find the methodology and results very interesting and believe they make an important contribution to urban planning by adapting cities to their specific contexts.

 Even so, I have some recommendations and comments that could improve the article:

 -Avoid using the word “elderly,” as it can be considered derogatory and ageist.

 -The hypothetical model seems overly complex; 18 hypotheses are too many, and they are not clearly reflected in the results, discussion, or conclusions.

 -Given the current scenario of climate change and extreme weather events in many parts of the world, thermal comfort and its effects on the health of older adults as vulnerable users should be considered. This could be acknowledged as one of the study’s limitations.

 -Avoid repeating in the text information already presented in tables and figures; for example, this occurs with Table 1 and the preceding text.

 -Figure 2: Add an abbreviation note explaining the meaning of each abbreviation (GPI, CEP, AUP, CFT, CNV, AST, ISL, IQL).

 -In the Results section, under the subtitle “Structural model,” there is a typo in the text: “increased socialization (p = 2.93; t = 0.00)”. I believe it should read t = 2.93 and p = 0.00. In the following sentence, “comfort has a positive significant relationship with increased physical activity level (t = 3.56; t = 0.00),” the second value should not be labeled as t.

 -More explanation is needed for Figure 3, as it appears to present the most important results, yet it lacks sufficient interpretation. I also suggest including abbreviations. Additionally, only seven hypotheses are mentioned under this figure.

 -Although 18 hypotheses are stated at the beginning of the paper, they are not validated or assessed in the results, discussion, or conclusions. It is important to include an analysis that explicitly evaluates these hypotheses, especially since a substantial portion of the introduction is devoted to describing the hypothetical model without a corresponding level of attention to its verification.

Is the work clearly and accurately presented and does it cite the current literature?

Yes

If applicable, is the statistical analysis and its interpretation appropriate?

Partly

Are all the source data underlying the results available to ensure full reproducibility?

Partly

Is the study design appropriate and is the work technically sound?

Yes

Are the conclusions drawn adequately supported by the results?

Partly

Are sufficient details of methods and analysis provided to allow replication by others?

Yes

Reviewer Expertise:

Urban planning, sustainability, age-friendly cities, urban health, accessibility, thermal comfort, environmental quality.

I confirm that I have read this submission and believe that I have an appropriate level of expertise to confirm that it is of an acceptable scientific standard, however I have significant reservations, as outlined above.

# Response

**Published:** 2026-01-08

**Authors:** Akshatha Rao

**Affiliations:** Manipal Academy of Higher Education, Manipal, Karnataka, India

Comments addressed from Reviewer 3: **Maria Teresa Baquero Larriva**,

 I find the methodology and results very interesting and believe they make an important contribution to urban planning by adapting cities to their specific contexts. Even so, I have some recommendations and comments that could improve the article:

 - **Avoid using the word “elderly,” as it can be considered derogatory and ageist.**

 We thank the reviewer for this observation.

 We have replaced all instances of the term “elderly” with **“**older adults **”** throughout the manuscript to ensure non-ageist language.

**The hypothetical model seems overly complex; 18 hypotheses are too many, and they are not clearly reflected in the results, discussion, or conclusions.**

 We retained all 18 hypotheses as each is theoretically essential. We have also clarified their connection to the results for better coherence.

**-Given the current scenario of climate change and extreme weather events in many parts of the world, thermal comfort and its effects on the health of older adults as vulnerable users should be considered. This could be acknowledged as one of the study’s limitations.**

 Thank you for highlighting this important point. We have added a sentence in the Limitations section acknowledging that: “Thermal comfort and climate-related factors were not included in this study, although they significantly influence walkability and the health of older adults, particularly in regions experiencing extreme weather conditions.”

**-Avoid repeating in the text information already presented in tables and figures; for example, this occurs with Table 1 and the preceding text.**

 We appreciate this suggestion. We have removed redundant descriptions that repeated information already shown in Table 1. The text now provides only a concise summary to avoid duplication and improve readability.

 Table 1 presents the sociodemographic characteristics of the sample. The respondents reflected a balanced gender distribution and were primarily in the 60–70-year age group. Most participants were married and held at least a bachelor’s degree. The income categories used in this study were: below poverty line (<3,565 USD/year), low income (3,565–7,150 USD), middle-income group 1 (7,150–14,300 USD), middle-income group 2 (14,300–21,450 USD), and high income (>21,450 USD). Overall, the sample represents a diverse cross-section of older adults.

**-Figure 2: Add an abbreviation note explaining the meaning of each abbreviation (GPI, CEP, AUP, CFT, CNV, AST, ISL, IQL).**

 We agree with the reviewer’s observation. An abbreviation note has been added below Figure 2 explaining each term clearly.

**-In the Results section, under the subtitle “Structural model,” there is a typo in the text: “increased socialization (p = 2.93; t = 0.00)”. I believe it should read t = 2.93 and p = 0.00. In the following sentence, “comfort has a positive significant relationship with increased physical activity level (t = 3.56; t = 0.00),” the second value should not be labeled as t**.

 Thank you for pointing out these typos. We have corrected the errors:

- “increased socialization (p = 2.93; t = 0.00)”
  → t = 2.93; p = 0.00
- “(t = 3.56; t = 0.00)”
  → t = 3.56; p = 0.00

We have also rechecked all statistical notation for accuracy.

 - **More explanation is needed for Figure 3, as it appears to present the most important results, yet it lacks sufficient interpretation. I also suggest including abbreviations.**

 Thank you for the comment. Abbreviations have now been added below Figure 3 for clarity. The explanation accompanying the figure has been retained, as the interpretation of all supported paths is already provided in detail within the Results and Discussion sections

**Additionally, only seven hypotheses are mentioned under this figure.**

 Thank you for the observation. Figure 3 displays only the supported hypotheses, which is why seven relationships are shown. The remaining hypotheses, which were not statistically significant, are reported separately in Table 4 and described in the Results section.

**-Although 18 hypotheses are stated at the beginning of the paper, they are not validated or assessed in the results, discussion, or conclusions. It is important to include an analysis that explicitly evaluates these hypotheses, especially since a substantial portion of the introduction is devoted to describing the hypothetical model without a corresponding level of attention to its verification**

 Thank you for the comment. All 18 hypotheses are validated in Table 4. To avoid redundancy, we did not include additional narrative evaluation, as the supported relationships are already explained in the Results and Discussion.

# Reviewer report: Reviewer response for version 2

**DOI:** 10.5256/f1000research.185485.r441027

**Published:** 2026-01-02

**Authors:** Rui Pedro Juliao

**Affiliations:** 1 Universidade Nova de Lisboa (NOVA-FCSH), Lisbon, Portugal

The paper addresses an up-to-date and relevant issue for city planning. After the first revision, the authors were able to fully improve it and respond accordingly to the suggestions that were made. The paper is well structured and written, the methodology is sound, and the results, as their interpretation, are relevant.

Is the work clearly and accurately presented and does it cite the current literature?

Yes

If applicable, is the statistical analysis and its interpretation appropriate?

Yes

Are all the source data underlying the results available to ensure full reproducibility?

Yes

Is the study design appropriate and is the work technically sound?

Yes

Are the conclusions drawn adequately supported by the results?

Yes

Are sufficient details of methods and analysis provided to allow replication by others?

Yes

Reviewer Expertise:

Territorial planning, Geographic Information Systems, Spatial analysis

I confirm that I have read this submission and believe that I have an appropriate level of expertise to confirm that it is of an acceptable scientific standard.

# Response

**Published:** 2026-01-04

**Authors:** Akshatha Rao

**Affiliations:** Manipal Academy of Higher Education, Manipal, Karnataka, India

Dear Dr. Rui Pedro Juliao,

 Thank you very much for your thorough review and for approving our manuscript. We sincerely appreciate the time, expertise, and care you have taken in evaluating the revised version of the paper.

 We are particularly grateful for your confirmation that the work meets the required scientific standards.

 As this paper forms a part of my PhD research, your approval is especially meaningful, and I truly appreciate your support in helping me reach this important academic milestone.

 Thank you once again.

 With kind regards,

 Akshatha Rao

 (on behalf of the authors)

# Reviewer report: Reviewer response for version 1

**DOI:** 10.5256/f1000research.177680.r371184

**Published:** 2025-04-25

**Authors:** Ana Margarita Larranaga

**Affiliations:** 1 Federal University of Rio, Porto Alegre, Brazil

The paper addresses an interesting and relevant topic. Below are some suggestions and points that I believe should be further detailed and explored to strengthen the overall clarity of the manuscript.

 Introduction

 -Please ensure that the constructs such as BEW, CMT, CNV, and AST are clearly defined and explained in the main text of the introduction. While these are mentioned in the abstract, they are not properly introduced or contextualized in the body of the paper.

 -I also recommend renaming these abbreviations using more intuitive terms to enhance readability and improve comprehension of the discussion and results. For example, instead of "CMT", use "Comfort", and instead of "AST", use "Aesthetics".Additionally, the review of existing literature could be significantly strengthened. I recommend providing a more in-depth discussion of previous studies in this area, including:where they were conducted, what specific aspects they analyzed,the key findings, and their limitations. This will help clarify the research gap and show how the current work contributes to advancing this line.

 .Hypothetical Model

 The current model presentation is confusing due to the excessive use of acronyms. I suggest renaming the variables with more descriptive terms. Also, the structure of the hypotheses would benefit from reorganization for clarity. In the diagram of the hypothetical model, please label each arrow with the corresponding hypothesis number (e.g., H1, H2...) and clearly state what relationship is being tested. This would greatly improve the reader's ability to follow and understand the logic of the model.

 Moreover, when citing studies that support the proposed hypotheses, please indicate where those studies were conducted. The urban context and local characteristics may significantly influence preferences and decision-making, and acknowledging this is essential.

 Methodology

 The method section requires more detail. Please clarify: How were participants recruited?, How long did the survey take to complete?, Were responses from different formats (e.g., manual and electronic) compared in any way, or were they merged directly for analysis?In the description of the study area, include relevant urban and socio-demographic characteristics, especially those that may influence walkability. A map and additional contextual details would be helpful for situating the study.

 Regarding the SEM analysis, the methodological procedures are not adequately described or justified. For example, the reliability and validity assessments appear only in the results section, but they should be introduced and explained in the methodology: Also,what software was used? What estimator was applied? Were Likert-scale variables treated as continuous or categorical? What model fit indices were used?

 Questionnaire Design

 I recommend including a table summarizing the questionnaire variables, indicating the construct each variable belongs to, the original source or basis for its inclusion, and any relevant literature that supports its use. This will greatly enhance the transparency and reproducibility of the instrument. As currently written, the long-form paragraph makes it difficult to follow the questionnaire structure.

 Discussion

 The discussion refers to existing literature but does not contextualize those findings in terms of where they occurred. It is important to consider how geographic, cultural, and infrastructural differences across study sites may explain variations in results or reinforce your findings.

Is the work clearly and accurately presented and does it cite the current literature?

Partly

If applicable, is the statistical analysis and its interpretation appropriate?

Partly

Are all the source data underlying the results available to ensure full reproducibility?

Partly

Is the study design appropriate and is the work technically sound?

Partly

Are the conclusions drawn adequately supported by the results?

Partly

Are sufficient details of methods and analysis provided to allow replication by others?

Partly

Reviewer Expertise:

Econometric modelling, Demand analysis, Acitve transport

I confirm that I have read this submission and believe that I have an appropriate level of expertise to confirm that it is of an acceptable scientific standard, however I have significant reservations, as outlined above.

# Response

**Published:** 2025-07-21

**Authors:** Akshatha Rao

**Affiliations:** Manipal Academy of Higher Education, Manipal, Karnataka, India

The paper addresses an interesting and relevant topic. Below are some suggestions and points that I believe should be further detailed and explored to strengthen the overall clarity of the manuscript.

 Introduction

 -Please ensure that the constructs such as BEW, CMT, CNV, and AST are clearly defined and explained in the main text of the introduction. While these are mentioned in the abstract, they are not properly introduced or contextualized in the body of the paper.

 -I also recommend renaming these abbreviations using more intuitive terms to enhance readability and improve comprehension of the discussion and results. For example, instead of "CMT", use "Comfort", and instead of "AST", use "Aesthetics".Additionally, the review of existing literature could be significantly strengthened. I recommend providing a more in-depth discussion of previous studies in this area, including:where they were conducted, what specific aspects they analyzed,the key findings, and their limitations. This will help clarify the research gap and show how the current work contributes to advancing this line.

**Answer**:

 Thank you very much for your valuable feedback. All suggested revisions have been carefully addressed and incorporated into the revised manuscript

 .Hypothetical Model

 The current model presentation is confusing due to the excessive use of acronyms. I suggest renaming the variables with more descriptive terms. Also, the structure of the hypotheses would benefit from reorganization for clarity. In the diagram of the hypothetical model, please label each arrow with the corresponding hypothesis number (e.g., H1, H2...) and clearly state what relationship is being tested. This would greatly improve the reader's ability to follow and understand the logic of the model.

 Moreover, when citing studies that support the proposed hypotheses, please indicate where those studies were conducted. The urban context and local characteristics may significantly influence preferences and decision-making, and acknowledging this is essential.

**Answer**:

 Thank you so much

 We appreciate the reviewer’s suggestion regarding the overuse of abbreviations in the model and manuscript. While abbreviations such as BEW, CMT, CNV, and AST were initially employed to maintain brevity and align with academic conventions in structural modeling, we acknowledge that their excessive use may hinder clarity for readers unfamiliar with these terms. Accordingly, we have revised the manuscript and the model diagram to replace the abbreviations with more intuitive and descriptive labels to enhance readability and ensure better comprehension of the constructs and their interrelationships.

 Methodology

 The method section requires more detail. Please clarify: How were participants recruited?, How long did the survey take to complete?, Were responses from different formats (e.g., manual and electronic) compared in any way, or were they merged directly for analysis?In the description of the study area, include relevant urban and socio-demographic characteristics, especially those that may influence walkability. A map and additional contextual details would be helpful for situating the study.

**Answer**:

 All the required details are provided in detail in the manuscript.

 The study area description and urban and socio-demographic characteristics with justification:  provided in the section “The study area”.

 Sampling and adequacy: provided in the section “Sampling procedure”

 Data collection duration, responses and formats: Provided in the section “Data collection Process”

 The recruitment process, the duration and the formats with the return rates, further ethical clearance details are also provided in the methodology.

 Regarding the SEM analysis, the methodological procedures are not adequately described or justified. For example, the reliability and validity assessments appear only in the results section, but they should be introduced and explained in the methodology: Also,what software was used? What estimator was applied? Were Likert-scale variables treated as continuous or categorical? What model fit indices were used?

**Answer**:

 Explained : In the section Data Analysis

 Questionnaire Design

 I recommend including a table summarizing the questionnaire variables, indicating the construct each variable belongs to, the original source or basis for its inclusion, and any relevant literature that supports its use. This will greatly enhance the transparency and reproducibility of the instrument. As currently written, the long-form paragraph makes it difficult to follow the questionnaire structure.

**Answer**:

 DOI added.

 Discussion

 The discussion refers to existing literature but does not contextualize those findings in terms of where they occurred. It is important to consider how geographic, cultural, and infrastructural differences across study sites may explain variations in results or reinforce your findings.

**Answer**:

 Thank you for the insightful comment. We acknowledge the importance of contextualizing our findings geographically and culturally. In response, we have revised the discussion section to include the specific urban and socio-cultural characteristics of Udupi, India, and how these may influence walkability outcomes in older adults.
